# Adipose tissue dysfunction increases fatty liver association with pre diabetes and newly diagnosed type 2 diabetes mellitus

**DOI:** 10.1186/s13098-016-0189-6

**Published:** 2016-11-11

**Authors:** Esteban Jorge-Galarza, Aida Medina-Urrutia, Rosalinda Posadas-Sánchez, Carlos Posadas-Romero, Guillermo Cardoso-Saldaña, Gilberto Vargas-Alarcón, Nacú Caracas-Portilla, Carmen González-Salazar, Margarita Torres-Tamayo, Juan Gabriel Juárez-Rojas

**Affiliations:** 1 Endocrinology Department, National Institute of Cardiology “Ignacio Chávez”, Juan Badiano No. 1, Col Sección XVI, Tlalpan, 14080 Mexico, Mexico; 2 Molecular Biology Department, National Institute of Cardiology “Ignacio Chávez”, Mexico, Mexico

**Keywords:** Impaired fasting glucose, Type 2 diabetes mellitus, Liver fat, Insulin resistance, Visceral fat

## Abstract

**Background:**

To evaluate the role of adipose tissue function on the association of fatty liver (FL) with impaired fasting glucose (IFG) or newly diagnosed type 2 diabetes mellitus (nT2D).

**Methods:**

In 1264 subjects, computed tomography was used to evaluate FL and elevated visceral adipose tissue (VAT). Fasting plasma glucose, <5.6, 5.6–6.9 and ≥7 mmol/l, were used to defined normoglycemic (NG), IFG or nT2D, respectively. Elevated free fatty acids, low serum adiponectin levels and adipose tissue insulin resistance (Adipo-IR), were used as markers of adipose tissue dysfunction.

**Results:**

Compared to NG subjects, those with IFG or nT2D had higher prevalence of FL and elevated VAT. FL was found to be independently associated with IFG and nT2D. Adipo-IR increased the association between FL and IFG [OR: 2.46 (95% I.C.: 1.73–3.49) to 5.42 (3.11–9.41)], whereas low adiponectin levels had a higher effect on the FL and nT2D association [OR: 4.26 (2.18–8.34) to 8.53 (2.96–24.55)].

**Conclusion:**

Fatty liver was independently associated with IFG and nT2D. Our results indicate for the first time, that adipose tissue dysfunction increases these associations.

**Electronic supplementary material:**

The online version of this article (doi:10.1186/s13098-016-0189-6) contains supplementary material, which is available to authorized users.

## Background

For many years adipose tissue was considered an organ of energy deposit and thermal insulation. However, this concept has changed during the last decades, and it is now clear that adipose tissue is a complex endocrine organ with high metabolic activity [1]. It has been postulated that dysfunction of adipose tissue begins when fat storage capacity of the subcutaneous compartment is diminished, which leads to fat accumulation in other organs and tissues [2]. Intrabdominal visceral adipose tissue (VAT) is one of the most important ectopic depots. Under insulin resistance conditions, VAT is a source of excessive release of free fatty acids (FFA) and inflammatory adipokines to the portal vein leading to hepatic fat accumulation, which in turn affects glucose and lipoprotein metabolism and contributes to the inflammatory process [3]. The total adipose tissue insulin resistance (Adipo-IR) may participate in this process by increasing triglycerides lipolysis [4, 5]. Dysfunction of adipose tissue is also characterized by low levels of adiponectin [6.]. In humans, adiponectin which is mainly synthesized by adipocytes, has been directly correlated with insulin sensitivity but inversely related with cardiovascular risk factors [7] and with hepatic fat content [8]. Because Adipo-IR, elevated FFA, and low adiponectin are abnormalities associated with adipose tissue excess, liver injury and related comorbidities, their presence could be considered as a marker of dysfunctional adipose tissue [4, 7].

Impaired fasting glucose (IFG) and type 2 diabetes mellitus have been associated with high total mortality risk [9]. VAT and hepatic fat are depots commonly associated with these two conditions [3, 10, 11] and it has been previously reported that VAT and fatty liver (FL) share similar effects on lipid and glucose metabolism [3, 12, 13]. Recent studies have proposed the hypothesis that compared to VAT, FL could have a greater impact on the development of metabolic derangements [14, 15]. Kantarzis et al. found that liver fat predicted glucose tolerance categories more strongly than VAT [15]. However, it is currently unknown whether functional features of adipose tissue could have a greater impact than its quantity on the association of liver fat with the risk of pre diabetes and type 2 diabetes mellitus. Therefore, the aim of the present study was to test the hypothesis that adipose tissue dysfunction (measured through FFA, adiponectin and Adipo-IR) participates on the association of liver fat with either IFG or newly diagnosed type 2 diabetes mellitus (nT2D), independently of the amount of VAT.

## Methods

### Study population

The study population included participants in the Genetics of Atherosclerotic Disease (GEA) study. The GEA study was designed to examine the genomic bases of coronary heart disease (CHD), and to assess relationships between traditional and emerging risk factors with clinical and subclinical atherosclerotic vascular disease in an adult Mexican population [16]. Briefly, a convenience sample from residents in Mexico City was recruited; this sample included non randomized, consecutive volunteers to form a control group of 1500 subjects aged 35 to 70 years. Patients with established premature CHD were consecutively selected from the outpatient clinic of the National Institute of Cardiology. Control participants without family history of premature CHD and no personal history of cardiovascular disease were recruited from Blood Bank donors, and through brochures posted in social service centers. Coronary patients and control subjects with history of renal, liver, thyroid or malignant disease, as well as those on treatment with corticosteroids, were excluded. Subjects with positive serology for viral hepatitis B and C, HIV, syphilis, and Chagas disease were also excluded.

**Fig. 1:**
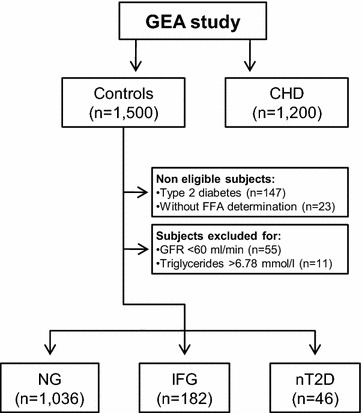
Flow chart of sample selection in the GEA study. Subjects of the control group in GEA study were stratified according to fasting plasma glucoses levels in normoglycemic (NG: < 5.6 mmol/l); impaired fasting glucose (IFG: ≥5.6 mmol/l but <7.0 mmol/l) or newly diagnosed type 2 diabetes (nTD2: ≥70.0 mmol/l). CHD: coronary heart disease, GFR: glomerular filtration rate, and FFA: free fatty acids

In the present study, we included 1264 participants from the original GEA control group (n = 1500). Subjects without FFA determination (n = 23) and with type 2 diabetes mellitus previously diagnosed (n = 147) were not eligible; whereas those with high plasma triglycerides (TG ≥ 6.78 mmol/l, n = 11) or low glomerular filtration rate (GFR ≤ 60 ml/min, n = 55) were excluded (Fig. 1). Participants were stratified as: (1) normoglycemic when fasting plasma glucose was <5.6 mmol/l (NG); (2) IFG when glucose levels were ≥5.6 mmol/l but <7.0 mmol/l; and (3) nT2D when glucose values were ≥70.0 mmol/l, using the cutoff points of the American Diabetes Association [17].

### Clinical assessment

All subjects were interviewed by a trained research staff and completed questionnaires to collect information pertaining to demographic characteristics, CHD history, medication, alcohol and tobacco use. All participants had a complete clinical examination. Height was measured to the nearest 0.1 cm using a rigid stadiometer, and weight was measured to the nearest 0.1 kg with the use of a balance scale. Body mass index (BMI) was calculated as weight in kilograms divided by height in meters squared. After a 10-min rest, blood pressure was measured 3 times; the average of the second and third blood pressure measurements was used for the analysis. Hypertension was defined as self-reported treatment with antihypertensive medications or a systolic blood pressure ≥140 mm Hg or diastolic blood pressure ≥90 mm Hg. Low adiponectin levels was defined as adiponectin values below 4 µg/ml [6]. Elevated VAT, elevated FFA and the presence of Adipo-IR were considered when their values were ≥75th percentile (VAT: 121 cm^2^ for women; 153 cm^2^ for men; FFA: 0.75 mmol/l for women, 0.61 mmol/l for men; Adipo-IR: 11.09 mmol/l·μU/l for women, 8.24 mmol/l μU/l for men). These cutoff points were obtained from a GEA study subsample of 101 men and 180 women without obesity, history of CHD and normal values of blood pressure, fasting glucose and lipids.

### Biochemical analysis

Venous blood samples were collected from subjects after a 12 h fasting and 20 min in a sitting position. Plasma glucose, TG, high density lipoprotein cholesterol (HDL-C) and FFA were measured using standardized enzymatic procedures (Roche Diagnostics GmbH, Mannheim, Germany). Accuracy and precision of lipid measurements in our laboratory are under periodic surveillance by the Center for Disease Control and Prevention service (Atlanta, GA, USA). Inter assay coefficients of variation were less than 6% for all of these assays. Low-density lipoprotein cholesterol (LDL-C) was estimated by using the De Long et al. method [18]; and GFR was estimated using the Cockroft-Gault formula [19]. High-sensitivity C-reactive protein (hsCRP) was determined by immunonephelometry on a BN Pro Spec nephelometer (Dade Behring, Marburg, Hesse, Germany), according to the manufacturer method. Plasma insulin concentrations were determined by a radioimmunometric assay (Millipore, St. Charles, Missouri, USA) and human total adiponectin was determined with a Quantikine ELISA kit (R&D Systems, Boston, Massachusetts, USA). Insulin resistance (IR) was estimated with the use of the homeostasis model assessment (HOMA-IR) [20] and the Adipo-IR was computed with a validated adipose tissue insulin resistance index (Adipo-IR = FFA [mmol/l] X insulin concentration [µU/l]) [4].

### Computed tomography study

CT is a validated method for measuring VAT [21] and FL [22]. In the present study, these measurements were obtained using a 64-slice scanner (Somatom Cardiac Sensation 64, Forcheim, Bavaria, Germany). To determine the liver and spleen attenuation, a single slice CT scan was obtained at the level of T11–T12 or T12–L1. Fatty liver was defined as a liver/spleen attenuation ratio lower than 1.0 [22]. To calculate the amount of total abdominal tissue (TAT) and VAT, a single slice scan was done at the level of L4–L5 and the area was expressed in cm^2^. Subcutaneous abdominal tissue (SAT) was calculated by subtracting the VAT from the TAT area.

### Statistical analysis

All variables were analyzed for normal distribution, using skewness and kurtosis. Data are expressed as mean ± standard deviation for variables with normal distribution, median (interquartile range) for skewed variables, and number of subjects (%) for categorical variables. Comparisons of means, medians and frequencies were made with ANOVA, Kruskal–Wallis and Chi squared tests, respectively. Bonferroni post hoc test was used for multiple pairwise comparisons. The relative contribution of fat depots or markers of adipose tissue function to IFG and nT2D was analyzed with the use of multinomial logistic regression analyses. To evaluate the role of adipose tissue function markers on the association of FL or elevated VAT, with IFG or nT2D, subjects with NG, IFG or nT2D were stratified by the presence of FL or elevated VAT alone, or its combination with each of the abnormal markers in a full adjusted multivariate model. All analyses were carried out with the software program STATA 12 (STATA CORP Texas, USA.). All p values <0.05 or confidence intervals 95% that excluded the unity, were considered statistically significant.

## Results

**Fig. 2:**
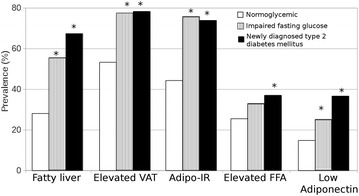
Prevalences of abnormal fat depots and markers of adipose tissue dysfunction in the three groups studied. Adipo-IR: adipose tissue insulin resistance; FFA: free fatty acids. *p < 0.005 vs normoglycemic group, Chi squared test was used

Metabolic characteristics of the studied groups are summarized in Table 1. Compared with the NG group, IFG and nT2D groups were older (51.7 ± 9, 54.2 ± 8 and 54.9 ± 9 years; respectively) and had a higer proportion of male subjects (51.8, 56 and 63%; respectively), as well as higher values of BMI, TG, fasting glucose, HOMA-IR, Adipo-IR, hsCRP, SAT, TAT and VAT, and lower values of HDL-C, adiponectin and liver/spleen attenuation ratio. Compared with IFG, nT2D group was higher in fasting plasma glucose and HOMA-IR, but lower in SAT and Adipo-IR. No differences were observed in physical activity, current smoking, and statin use among different groups (data not shown). Compared with NG, subjects with IFG and nT2D had higher prevalences of FL, elevated VAT, Adipo-IR, elevated FFA and low adiponectin. No differences were found between subjects with glucose abnormalities (IFG and nT2D) (Fig. 2).

**Table 1:** Metabolic characteristics of the studied groups

	Normoglycemic n = 1036	Impaired fasting glucose n = 182	Newly diagnosed type 2 diabetes mellitus n = 46	p trend
Age (years)	51.7 ± 9	54.2 ± 8*	54.9 ± 9	<0.001
Gender (male)	499 (51.8%)	102 (56%)	29 (63%)*	0.017
BMI (kg/m^2^)	28.2 ± 4	30.2 ± 4*	30.4 ± 5*	<0.001
Alcohol consumption (>30 gr/day)	18 (1.7%)	3 (1.7%)	2 (4.3%)	0.610
Hypertension (%)	209 (20)	55 (30)*	12 (26)	<0.001
LDL-C (mmol/l)	3.08 ± 0.8	3.21 ± 0.9	3.10 ± 0.8	0.195
HDL-C (mmol/l)	1.16 (0.96–1.40)	1.05 (0.88–1.24)*	1.04 (0.85–1.16)*	<0.001
Triglycerides (mmol/l)	1.60 (1.21–2.18)	2.01 (1.45–2.73)*	2.29 (1.67–3.13)*	<0.001
Fasting glucose (mmol/l)	4.89 (4.6–5.2)	5.77 (5.7–6.1)*	8.49 (7.7–12.2)*^†^	<0.001
HOMA-IR	3.4 (2.49–4.89)	6.16 (4.6–8.1)*	8.98 (6.4–12)*^†^	<0.001
hsCRP (nmol/l)	14.0 (7.52–28.6)	21.8 (9.5–38.0)*	21.8 (10.5–36.2)*	<0.001
Adipo-IR (mmol/l·μU/l)	8.8 (5.7–13.1)	13.3 (9.2–20)*	12.3 (8.9–17.9)*^†^	<0.001
Free fatty acids (mmol/l)	0.55 (0.43–0.69)	0.57 (0.43–0.69)	0.59 (0.52–0.76)*	0.0312
Adiponectin (µg/ml)^a^	8.3 (5.1–13)	6.7 (3.9-10–2)*	6.4 (2.8–9.2)*	<0.001
Subcutaneous adipose tissue (cm^2^)	282 (212–360)	307 (250–384)*	297 (2–405)^†^	0.005
Total adipose tissue (cm^2^)	428 (3–531)	491 (424–587)*	502 (403–607)*	<0.001
Visceral adipose tissue (cm^2^)	143 (105–184)	176 (139–229)*	177 (139–229)*	<0.001
Liver to spleen attenuation ratio	1.12 (0.96–1.22)	0.96 (0.75–1.11)*	0.89 (0.68–1.01)*	<0.001

Values are expressed as mean ± standard deviation, median (interquartile range) or number of subjects (%)

*HDL*-*C* high density lipoprotein cholesterol, *LDL*-*C* low density lipoprotein cholesterol, *HOMA*-*IR* homeostasis model assessment of insulin resistance

* p < 0.05 vs normoglycemic

^†^ p < 0.05 vs impaired fasting glucose

^a^n = 985 for normoglycemic group, n = 167 for impaired fasting glucose group and n = 41 for newly diagnosed type 2 diabetes mellitus group

Logistic regression analysis was used to evaluate the individual associations of fat depots and markers of adipose tissue function with IFG and nT2D (Table 2). Results showed that independent of age and gender, IFG and nT2D subjects were more likely to have FL, elevated VAT, Adipo-IR, and low adiponectin levels (model 1). In a fully adjusted model (model 2), FL but not VAT, remained associated with IFG and with nT2D. Among the adipose tissue function markers, Adipo-IR was associated with both glucose abnormalities, whereas low adiponectin levels were associated only with nT2D. To confirm the results, subjects from IFG or nT2D groups were matched by age and gender with control subjects that did not have obesity and metabolic syndrome. The fully adjusted model showed that compared with control subjects without obesity and metabolic syndrome, those with IFG or nT2D had higher risk of fatty liver disease [OR: 2.35 (C.I. 95%: 1.32–4.16) and 3.78 (1.67–8.53); respectively].

**Table 2:** Association of fat depots and markers of adipose tissue dysfunction with impaired fasting glucose and newly diagnosed type 2 diabetes mellitus

Abnormalities	Normoglycemic	Impaired fasting glucose	Newly diagnosed type 2 diabetes mellitus
	Odd ratio (95% I.C)	Odd ratio (95% I.C)	Odd ratio (95% I.C)
Model 1	Reference values		
Fatty liver	1	3.36 (2.43–4.65)	5.68 (3.01–10.69)
Elevated VAT	1	2.79 (1.91–4.05)	2.84 (1.38–5.82)
Adipo-IR	1	3.80 (2.65–5.47)	2.15 (1.54–3.02)
Low adiponectin	1	1.84 (1.22–2.77)	3.29 (1.64–6.58)
Elevated FFA	1	1.37 (0.97–1.94)	1.62 (0.87–3.01)
Model 2			
Fatty liver^a^	1	2.46 (1.73–3.49)	4.26 (2.18–8.25)
Elevated VAT^b^	1	1.37 (0.89–2.11)	1.09 (0.49–2.44)
Adipo-IR	1	2.69 (1.82–3.96)	2.15 (1.06–4.37)
Low adiponectin	1	1.46 (0.96-2.22)	2.54 (1.26–5.12)
Elevated FFA	1	1.29 (0.91-1.85)	1.51 (0.80–2.85)

Model 1 adjusted for age and gender

Model 2 adjusted for age, gender, body mass index, high density lipoprotein cholesterol and triglycerides

*VAT* visceral abdominal tissue, *Adipo*-*IR* adipose tissue insulin resistance, *FFA* free fatty acids

^a^Additional adjustment for elevated VAT

^b^Additional adjustment for fatty liver

To evaluate the potential effect of adipose tissue dysfunction on the association of FL with IFG or nT2D, we assessed the effect of FL alone and also the effect of FL plus each one of the adipose tissue function markers (Table 3). These analyses showed that the presence of Adipo-IR had the strongest effect on the association between FL and IFG, followed by low adiponectin levels and elevated FFA. Conversely, the presence of low adiponectin had a significantly higher effect on the FL and nT2D association, followed by Adipo-IR and elevated FFA. In the paired-matched sub-analysis, the effect of low adiponectin on the association of FL with glucose categories was attenuated, and the effect of the other adipose tissue markers on the association of FL with glucoses abnormalities did not change (Additional file 1: Table S1). Although elevated VAT was not independently associated with IFG or nT2D, a similar analysis was performed to evaluate the effect of adipose tissue dysfunction on the association between elevated VAT and glucose abnormalities. The results showed that Adipo-IR was the only marker that increased the association of elevated VAT with IFG [2.99 (1.62–5.55), p < 0.05].

**Table 3:** Combined association of fatty liver and markers of adipose tissue dysfunction with impaired fasting glucose and newly diagnosed type 2 diabetes mellitus

Abnormalities	Normoglycemic	Impaired fasting glucose	Newly diagnosed type 2 diabetes mellitus
	Reference values	Odds ratio (95% I.C)	Odds ratio (95% I.C)
Fatty liver	1	2.46 (1.73–3.49)	4.26 (2.18–8.34)
Fatty liver + Adipo-IR	1	5.42 (3.11–9.41)	6.81 (2.29–20.23)
Fatty liver + low adiponectin	1	3.89 (2.11–7.17)	8.53 (2.96–24-55)
Fatty liver + elevated FFA	1	2.66 (1.56–4.57)	4.99 (2.04–12.19)

Model adjusted for age, gender, body mass index, high density lipoprotein cholesterol, triglycerides and elevated VAT

*Adipo-IR* adipose tissue insulin resistance, *VAT* visceral adipose tissue, *FFA* free fatty acids

## Discussion

Previous studies have shown that both, visceral and hepatic fat depots are associated with increased risk of IFG and type 2 diabetes mellitus [11, 15, 23]. Recently, it has been proposed that compared to VAT, FL could have a greater impact on the development of metabolic derangements. Moreover, hepatic fat accumulation has been associated to dysfunctional adipose tissue, which is characterized by Adipo-IR, elevated FFA, and low adiponectin plasma levels [4, 24]. The results of the present study confirm these associations and show that Adipo-IR and low adiponectin could have an important role in the association of FL with IFG or nT2D. Our data also show that the combined effect of FL plus dysfunctional adipose tissue on IFG and nT2D is independent of VAT. These findings extend the knowledge about adipose tissue influence on the association of fat depots and glucose metabolic abnormalities.

Pre diabetes is a condition where early abnormalities in glucose metabolism are present but elevation in blood glucose is below cutoff point for establishing the diagnosis of type 2 diabetes mellitus [9]. IFG is a state of pre diabetes, closely associated with type 2 diabetes mellitus and is originated by multiple risk factors. The present and previous reports [15, 25, 26] have shown that NAFLD is independently associated with pre diabetes. However, in a very recent study, Ming et al. found no association between fatty liver and pre diabetes [27]. The contrasting results could be explained by differences in study design, sample size, ethnicity, studied population and pre diabetes definition. It is important to consider that the correlation between visceral and liver fat makes it difficult to discern the relative contribution of each fat depot on the risk of glucose abnormalities. Recent data has shown an independent association of VAT with the presence of type 2 diabetes mellitus [10, 11], but there are also previous reports indicating that liver fat content was associated with type 2 diabetes mellitus independently of VAT [28, 29]. Fabbrini et al. compared subjects with different VAT volume paired by liver fat content and found no differences regarding metabolic abnormalities of insulin resistance. On the other hand, when comparing subjects with different liver fat content but similar VAT, they found that metabolic alterations and insulin resistance were explained by intra hepatic fat content [14]. Consistent with these findings, the results of the present study showed that FL is associated with a higher probability of having nT2D, independent of traditional risk factors and elevated VAT (Table 2). Together these results are in line with the proposed hypothesis that in some cases, the reported association between VAT and derangements in glucose metabolism may be explained through the close relationship between VAT and liver fat content [14]. However, there are also data suggesting that obesity and FL may act through different mechanisms to increase the risk of type 2 diabetes mellitus [30].

Several studies have postulated that dysfunctional adipose tissue, favors the release of FFA to the portal circulation, and then to the liver where they accumulate and induce hepatic steatosis, inflammation, insulin resistance and 2 diabetes mellitus [3, 4, 31]. Dysfunctional adipocytes also show an abnormal anti-inflammatory response, characterized by lower synthesis and secretion of adiponectin. Low levels of adiponectin have been associated with insulin resistance, type 2 diabetes mellitus and FL [2, 6, 7]. Lomonaco et al. showed that a modest increase in Adipo-IR is associated with low adiponectin plasma levels, dyslipidemia, hepatic and muscle insulin resistance and hepatic steatosis. Similarly, the results of the present study showed that subjects with IFG and nT2D have higher visceral and hepatic fat content, as well as lower levels of adiponectin and higher levels of Adipo-IR. Moreover, these results indicate that in subjects with FL, both Adipo-IR and low adiponectin, respectively increase 110 and 50%, the probability of having IFG. The risk of having nT2D was higher in subjects with FL plus low adiponectin (84%) or FL plus Adipo-IR (48%). Adipo-IR was the only variable that significantly increased the association of elevated VAT with IFG (117%). The finding that Adipo-IR increased the risk of IFG in subjects with FL or elevated VAT, suggests that lipolysis induced by insulin resistance may be a key mediator in the early stages of metabolic derangements in subjects with ectopic fat excess. Our findings are further supported by the recent findings showing that liver fat accumulation is associated with decreased branched-chain amino acids catabolism, which suggest that adipose tissue dysfunction may play a key role in the systemic nature of NAFLD pathogenesis (32). On the other hand, the association of low adiponectin with nT2D found in the present study could reflect more advanced stages of metabolic alterations where inflammation plays a more definitive role [33, 34]. Furthermore, it has been previously reported that adiponectin expression is decreased by 20–40% in the presence of NAFLD, and plasma adiponectin concentrations are inversely related to hepatic fat content in patients with type 2 diabetes mellitus [8]. All these data suggest that adiponectin may also play an important pathophysiological role in the metabolic abnormalities associated with liver injury. Although the cause of total adipose tissue dysfunction, is not fully understood, hypoxia [35], PPAR gamma activation [2], defects in fatty acids oxidation [36], down-regulation of branched-chain amino acids catabolism [32], and genetic predisposition [37] could be involved.

The present study has some limitations. First, causality cannot be determined due to the cross-sectional nature of the analyses. Second, the presence of subjects with glucose intolerance could not be ruled out in the population studied, however, similar to our observations, previous studies have found that IFG is mainly associated with derangements in hepatic insulin sensitivity [38]. Third, the diagnosis of FL was not confirmed with hepatic biopsy specimens; however, significant correlations have been reported between imaging attenuation and the histology grade of steatosis [39]. Although subjects with viral hepatitis B and C, human immunodeficiency virus, syphilis and Chagas disease were excluded from the analyses, other causes of fatty liver such as viral hepatitis A, D, E and G, autoimmune hepatitis, metabolic liver disease or genetic factors were not excluded. We only analyzed the impact of PNPLA3 genotypes, and found no association between PNPLA3 and glucose metabolism abnormalities (data not shown). However, other fatty liver associated genotypes such as TM6SF2 variants were not explored. Therefore, their influence on the results cannot be ruled out. Fourth, our study included a Mexican-mestizo population; therefore, our findings may not be generalized to other ethnic groups. Finally, due to the small number of subjects with nT2D, these findings should be interpreted with caution and considered as hypotheses generating. These results should be confirmed by studies with a larger number of subjects.

## Conclusion

Our results show that FL is independently associated with IFG and nT2D. Furthermore, this study suggests that Adipo-IR and low levels of adiponectin may increase the association of FL with IFG and nT2D. Even though the volume of VAT was not independently associated with higher type 2 diabetes mellitus risk in this population, the presence of Adipo-IR significantly increased the risk of IFG, in subjects with elevated VAT.

## Additional file

**Additional file 1: Table S1.** Combined association of fatty liver and markers of adipose tissue dysfunction with the risk of IFG and nT2D in the paired-matched subpopulation.

## Abbreviations

FL: fatty liver
IFG: impaired fasting glucose
nT2D: newly diagnosed type 2 diabetes mellitus
VAT: visceral adipose tissue
Adipo-IR: adipose tissue insulin resistance
FFA: free fatty acids
