# Supplementary material for: Adipose tissue dysfunction increases fatty liver association with pre diabetes and newly diagnosed type 2 diabetes mellitus
Source: Diabetol Metab Syndr. 2016 Nov 11;8:73. doi: 10.1186/s13098-016-0189-6 (PMC5105292; doi:10.1186/s13098-016-0189-6)
Supplement: Supplementary file 1 — Additional file 1: Table S1. Combined association of fatty liver and markers of adipose tissue dysfunction with the risk of IFG and nT2D in the paired-matched subpopulation. [file 13098_2016_189_MOESM1_ESM.doc]

**Supplementary Table.** Combined association of fatty liver and markers of adipose tissue dysfunction with the risk of IFG and nT2D in the paired-matched subpopulation.

|  | NG | IFG | nT2D |
| --- | --- | --- | --- |
| Abnormalities | Reference | Odds ratio (95% I.C) | Odds ratio (95% I.C) |
| Fatty liver | 1 | 2.35 (1.32-4.16) | 3.78 (1.67-8.53) |
| Fatty liver + Adipo-IR | 1 | 4.48 (1.95-10.30) | 5.29 (1.47-19.07) |
| Fatty liver + Low adiponectin | 1 | 2.21 (0.78-6.22) | 4.49 (1.20-16.83) |
| Fatty liver + elevated FFA | 1 | 3.40 (1.34-8.67) | 5.95 (1.83-19.42) |

NG: normoglycemic; IFG: impaired fasting glucose; nT2D: newly diagnosed type 2 diabetes mellitus; Adipo-IR: adipose tissue insulin resistance; VAT: visceral adipose tissue; FFA: free fatty acids.

Model adjusted for age, gender, body mass index, high density lipoprotein cholesterol, triglycerides, and elevated VAT.
